# Artificial intelligence in coronary computed tomography angiography: Demands and solutions from a clinical perspective

**DOI:** 10.3389/fcvm.2023.1120361

**Published:** 2023-02-16

**Authors:** Bettina Baeßler, Michael Götz, Charalambos Antoniades, Julius F. Heidenreich, Tim Leiner, Meinrad Beer

**Affiliations:** ^1^Department of Diagnostic and Interventional Radiology, University Hospital Würzburg, Würzburg, Germany; ^2^Division of Experimental Radiology, Department for Diagnostic and Interventional Radiology, University Hospital Ulm, Ulm, Germany; ^3^British Heart Foundation Chair of Cardiovascular Medicine, Cardiovascular Medicine, University of Oxford, Oxford, United Kingdom; ^4^Department of Radiology, Mayo Clinic, Rochester, MN, United States; ^5^Department of Radiology, University Medical Center Utrecht, Utrecht, Netherlands; ^6^Department for Diagnostic and Interventional Radiology, University Hospital Ulm, Ulm, Germany

**Keywords:** cardiac computed tomography, artificial intelligence, clinical workflow, machine learning, deep learning, radiomics, coronary computed tomography angiography

## Abstract

Coronary computed tomography angiography (CCTA) is increasingly the cornerstone in the management of patients with chronic coronary syndromes. This fact is reflected by current guidelines, which show a fundamental shift towards non-invasive imaging - especially CCTA. The guidelines for acute and stable coronary artery disease (CAD) of the European Society of Cardiology from 2019 and 2020 emphasize this shift. However, to fulfill this new role, a broader availability in adjunct with increased robustness of data acquisition and speed of data reporting of CCTA is needed. Artificial intelligence (AI) has made enormous progress for all imaging methodologies concerning (semi)-automatic tools for data acquisition and data post-processing, with outreach toward decision support systems. Besides onco- and neuroimaging, cardiac imaging is one of the main areas of application. Most current AI developments in the scenario of cardiac imaging are related to data postprocessing. However, AI applications (including radiomics) for CCTA also should enclose data acquisition (especially the fact of dose reduction) and data interpretation (presence and extent of CAD). The main effort will be to integrate these AI-driven processes into the clinical workflow, and to combine imaging data/results with further clinical data, thus - beyond the diagnosis of CAD- enabling prediction and forecast of morbidity and mortality. Furthermore, data fusing for therapy planning (e.g., invasive angiography/TAVI planning) will be warranted. The aim of this review is to present a holistic overview of AI applications in CCTA (including radiomics) under the umbrella of clinical workflows and clinical decision-making. The review first summarizes and analyzes applications for the main role of CCTA, i.e., to non-invasively rule out stable coronary artery disease. In the second step, AI applications for additional diagnostic purposes, i.e., to improve diagnostic power (CAC = coronary artery classifications), improve differential diagnosis (CT-FFR and CT perfusion), and finally improve prognosis (again CAC plus epi- and pericardial fat analysis) are reviewed.

## 1. Introduction

Despite major advances in prevention, diagnosis, and treatment over the last decades, cardiovascular disease remains by far the number one cause of morbidity and mortality for both men and women worldwide, accounting for over 17 million deaths per year (1). The number of annual global deaths is expected to rise up to 24 million by 2030 (2, 3), thus imposing a huge challenge to global healthcare systems.

Although invasive coronary angiography currently remains the standard for the diagnosis and treatment of coronary artery disease (CAD), non-invasive imaging becomes increasingly important in the diagnostic workup. Especially cardiac computed tomography (CT), namely CT coronary angiography (CCTA) will become increasingly important to rule out CAD within the next few years, current guidelines having pushed CCTA to the frontline in the assessment of stable coronary syndromes (4, 5). Consequently, the radiologists’ workload will experience further increase, and questions arise regarding the handling of the increasing workload, the availability, expertise, radiation exposure, and the reimbursement of CCTA.

Artificial intelligence (AI), including Machine Learning (ML) and Deep Learning (DL) as well as related techniques extracting quantitative information from radiological images such as radiomics might be ideally suited to solve these challenges. Possible improvements by AI for CCTA encompass data acquisition, image post-processing, and interpretation, the latter in the way of decision support systems (6), as well as risk stratification. By this, AI in CCTA has the potential to improve patient management through an increase in the accuracy and efficiency of diagnosis and treatment planning.

AI can be used to automatically identify and characterize abnormalities in CCTA scans, such as calcified or non-calcified plaques, stenoses (narrowing of the coronary artery), and other features of interest. AI can also be used to predict the likelihood of future cardiovascular events, based on the characteristics of the abnormalities present in the CT images. This can help clinicians to identify patients who may be at higher risk and take steps to prevent or mitigate these events.

**Figure 1:**
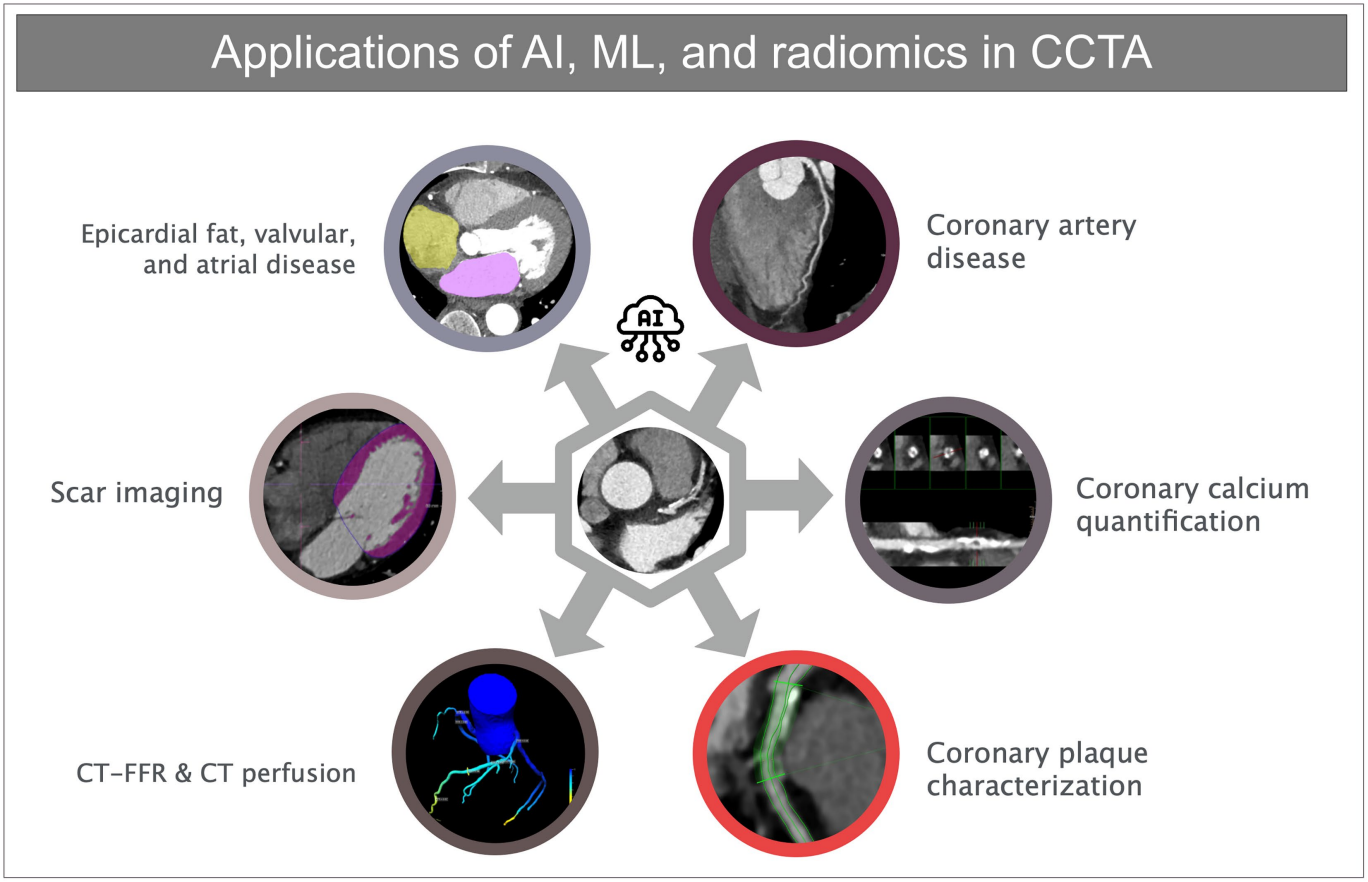
Overview of different applications of artificial intelligence, machine learning, and radiomics in coronary CT angiography.

A rapidly increasing number of publications deal with the role of AI in CCTA (Figure 1). However, some publications are orchestrated in the shape of great debates pushing pros and cons forward (7, 8), or concentrate on single aspects of CCTA such as imaging the coronary tree (9, 10), CT fractional flow reserve (FFR) (11, 12), epicardial adipose tissue (EAT) (13), or multimodality imaging machine learning (ML) applications (14). Only some reports so far span the horizon from basics to clinical practice and concentrate on future applications (15–17).

The aim of this review is to present a holistic overview of AI applications in CCTA (including radiomics) under the umbrella of clinical workflows and clinical decision-making. The review first summarizes and analyzes applications for the main role of CCTA, i.e., to non-invasively rule out stable coronary artery disease. In the second step, AI applications for additional diagnostic purposes, i.e., to improve diagnostic power (CAC = coronary artery classifications), improve differential diagnosis (CT-FFR and CT perfusion), and finally improve prognosis (again CAC plus epi- and pericardial fat analysis) are reviewed.

Following a short introduction to the technical basics of ML and radiomics, three parts/steps will be presented for each topic. Firstly, applications that are already integrated into daily clinical workflows (but might not be recognized as already “active”), secondly, applications that are at the barrier to clinical application, and thirdly, applications that might be realized in the near future and are highly awaited.

## 2. Technical basics

### 2.1. Machine learning basics

ML mimics humans’ most valuable skill, the ability to learn and improve from data. Based on previously gathered information, generic models detect patterns and use them to infer values for new data (18). This allows for solving various tasks without the need to explicitly program problem-specific algorithms.

**Figure 2:**
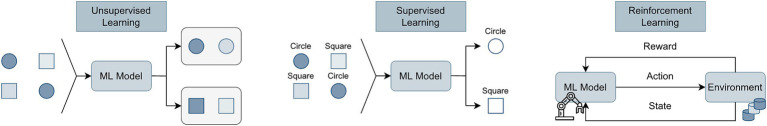
Different types of Machine Learning. Unsupervised Learning detects patterns. Supervised learning uses direct annotation of the data, while reinforcement learning interacts with an environment and learns using rewards for individual actions to solve specific tasks.

Depending on the degree and type of supervision, three subtypes of ML can be distinguished: supervised learning, unsupervised learning, and reinforcement learning (Figure 2) (19). Unsupervised learning aims to analyze data and detect hidden structures and correlations without solving a specific task or predicting endpoints. The other two types are used to solve specific tasks (like predicting endpoints) and differ in the type of supervision. Supervised learning reproduces given labels from the data, with the label types and completeness depending on the actual problem. In contrast, direct labels are avoided for reinforcement learning, and positive and negative feedback from the environment is utilized instead. Independently from the subtype, the final performance of an ML solution depends on the complexity, quality, and amount of available data. In contrast, reinforcement learning utilizes positive and negative feedback from the environment instead. An example of this is learning to move a robot, where the correctness of each movement depends on the final result which can then be used as feedback.

Independently from the subtype, the final performance of an ML solution depends on the complexity, quality, and amount of available data.

A recently very successful subsection of ML is Deep Learning (DL). DL models consist of multiple artificial neurons, typically organized in layers. The high flexibility in the connection of neurons or layers allows the adaptation to different tasks and data representations (18). This allows not only to address a wide range of applications but also the combination of data representation learning and task learning, efficiently enabling the usage of raw data and omitting the need for hand-crafted features (20). As a consequence, the advent of DL significantly increased the accuracy and applicability of learning-based solutions, especially in image-based domains (21). Here, Convolutional Neural Networks (CNNs), which incorporate the idea of learning convolutional filters for feature learning proved to be especially successful, like the well-known UNet (22) and the derived UNETR architecture (23).

Besides network architecture, the performance of a DL model is also defined by other parameters like the training algorithm or the loss/cost function. The networks are usually iteratively improved by reducing the loss within parts of the training data, the so-called batches. This process is repeated for the next epoch, once all data has been used. The loss function defines the task of the network by rating the quality of the output and possibly includes additional measures for enforcing sparsity, considering unlabeled data, or ensuring robustness. Together with the architecture these configuration parameters are responsible for the performance of a deep learning solution (24).

### 2.2. Radiomics basics

Radiomics is another technical principle to deal with imaging data, although there is some overlap with the field of ML illustrated above. Radiomic analyses use the data which can be extracted from the distribution and/or co-occurrence of pixel grey levels encoded in medical images of all modalities. It thereby allows for quantitative image analysis and the potential extraction of novel imaging biomarkers to get more information about tissue structure and the underlying histopathological phenotype, which otherwise would be not accessible through pure visual image inspection (25–27).

Radiomics allows quantifying differences in image intensity, shape, or texture (28, 29) and thus might be able to overcome the usual subjective nature of image interpretation. The extracted information is then processed *via* ML (since innumerable radiomic features can be extracted nowadays, which makes radiomics a “big data” approach) to enhance the existing clinical data for improved diagnosis, prognosis, or assessment of treatment response.

**Figure 3:**
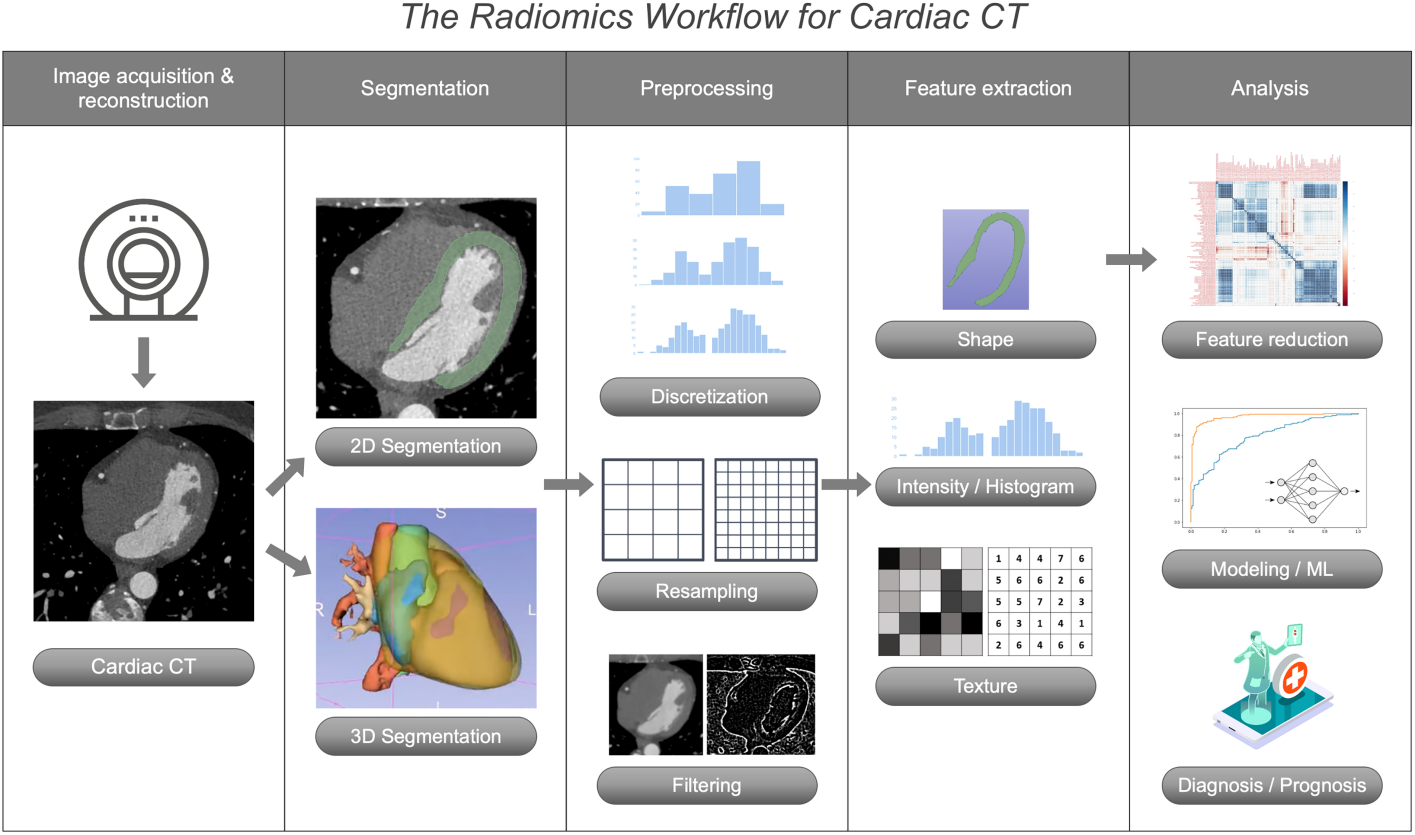
The Radiomics workflow for coronary CT angiography.

Radiomic analyses are performed *via* a step-wise workflow including patient scheduling and image acquisition/reconstruction, image segmentation and processing, and finally extraction of radiomics features from different feature matrices, consecutive data analysis, and building of predictive models for improved diagnostic or prognostic assessment, which is illustrated in Figure 3.

## 3. Clinical applications of machine learning, artificial intelligence, and radiomics for CCTA

### 3.1. The mainstay of CCTA: Coronary artery disease

CCTA is likely to become the future standard method to exclude stable CAD (5). Given the associated considerable increase in workload, there is a high need for automation along the entire imaging workflow. Potential applications for AI arise in several ways: (i) in data acquisition including patient scheduling and preparation, (ii) in data post-processing, and (iii) in data interpretation.

While AI-based approaches are established and available or at least proposed for data acquisition, these are largely lacking for patient preparation. AI-based models for automatic detection of ECG signals (ideally integrated into the patient table) as well as suggestions for scan planning based on patient geometry (involving 3D cameras) are already at hand. Apart from that, ML could be used to prove proper indication for CT examination and facilitate patient scheduling using cluster algorithms as well as automated reminders to reduce no-shows. Immediate exam preparation includes the appropriate decision of correct premedication (which includes both the administration of beta blockers and nitroglycerin) as well as the evaluation of respective contraindications since these are essential to achieve optimal scan conditions. Until now, no robust AI model has been proposed to support these crucial preparation steps.

A wide variety of protocols are available for data acquisition. Their selection depends on the examination conditions, especially the heart rate. Today’s modern scanners already offer AI-based protocol suggestions. However, these do not yet fully take into account patient geometry (e.g., the presence of obesity). Comprehensive protocol proposals are desirable, which might offer prospective as well as retrospective data acquisition, optimized scan and reconstruction algorithms for dose reduction (e.g., type of iterative or AI-based reconstruction), AI-based noise reduction, or improved spatial and temporal resolution. Even state-of-the-art dual-source CT scanners only allow for a minimum temporal resolution of 68 ms, far below what is necessary to allow optimal coronary image acquisition. Looking at modern spectral CT scanners, AI-based algorithms that consider the spectral information itself as additional information (e.g., as a 4th dimension of the dataset) currently are still missing. This will change with the help of AI-based algorithms. Conversely, Lyu et al. have demonstrated the feasibility of estimating dual-energy information from conventional single-energy CT datasets utilizing a combination of fully sampled low-energy data and a single-view high-energy projection (30).

AI-based algorithms are already available for largely automated 2D and 3D reformations of the coronary tree. However, manual interaction of technicians and/or radiologists is still necessary regarding the representation in standard coronal and sagittal views. AI-based algorithms now allow the automated generation of curved multiplanar reformations or so-called stretched coronaries.

Recently, the first reports have been published on the AI-based automated detection of coronary stenoses in CCTA (31–33). In a 2021 retrospective, multi-center study, the diagnostic accuracy and generalizability of an established DL-based fully automated algorithm in detecting coronary stenosis on CCTA has been shown to perform non-inferior to expert readers in detecting coronary stenoses ≥50% (34). In the vessel-based evaluation, the DL algorithm had a higher sensitivity (65.7%) and negative predictive value (NPV) (78.8%) than human expert readers. In 2022, a substudy from the CREDENCE (Computed TomogRaphic Evaluation of Atherosclerotic DEtermiNants of Myocardial IsChEmia) trial retrospectively analyzed a Food and Drug Administration–cleared cloud-based software that performs AI-enabled coronary segmentation, lumen and vessel wall determination, plaque quantification and characterization, and stenosis determination in order to detect the presence of ≥50% and ≥ 70% stenosis, respectively (32). The authors could demonstrate rapid and accurate identification and exclusion of high-grade stenosis with a close agreement to blinded, core lab–interpreted quantitative coronary angiography (per-patient sensitivity, specificity, PPV, NPV, and accuracy of 94, 68, 81, 90, and 84%, respectively, for ≥50% stenosis, and of 94, 82, 69, 97, and 86%, respectively, for detection of ≥70% stenosis).

While current CCTA reporting is mainly based on visual estimation of coronary stenosis grade, Hong et al. reported a DL model to accurately and quantitatively measure the stenosis grade in diseased coronary segments in comparison to an expert reader (35). The future use of such models thus might enable a faster and more accurate quantitative assessment of coronary stenoses.

The recent update of the CAD-Reporting and Data System (CAD-RADS) 2.0 (36) underlines the feasibility of standardized data reporting including follow-up instructions. While CAD-RADS 2.0 is relatively robust to subjective reader bias, proposed AI models predicted the CAD-RADS level in close agreement with expert readers (37). Regarding the follow-up recommendations, AI models have surpassed CAD-RADS in the discrimination of patients with and without subsequent adverse events (38).

The association of radiomics- and AI-derived features with other risk factors in CAD in the long-term view might be of particular interest. Kolossvary et al. performed a study using radiomics-based precision phenotyping, indicating that conventional risk factors, cocaine use, and HIV infection each had different effects on CT angiographic morphologic changes in coronary atherosclerosis over a follow-up period of 4 years (39). Eslami et al. trained a radiomics-based ML model to predict cardiovascular events in the Framingham Heart Study (40). In addition, several studies have been published over the past few years highlighting the potential of ML (using or not using radiomics) for outcome prediction in CAD (41–44).

### 3.2. Coronary calcium quantification

From a clinical perspective, coronary calcium detection and quantification both have a checkered history. Initially promoted as one of the essential building blocks for detection and prognostication in CAD (45), the importance of coronary calcium quantification was subsequently cast into doubt. In recent years, a renaissance occurred. However, it is still controversial whether coronary calcification can be used as a gatekeeper. Certainly, lipid-rich plaques cannot be detected without contrast administration. However, studies show that a threshold of an Agatston score of 400 is associated with a high significance of cardiac morbidity and mortality.

Conventionally, coronary calcium scoring is performed using high-pitch ECG-gated scanning to enable imaging within one breath-hold. Automated detection of calcified plaques using these dedicated scan protocols is well-established (46) but often needs manual revision to generate a robust Agatston score for risk stratification.

Since a high number of native chest CT scans are obtained without using ECG-gating or fixed tube voltage, AI models that can predict the Agatston score on these scans would be desirable. Amongst others, a promising model has been proposed that can generalize coronary artery calcium scores in different native CT scans with and without ECG gating, yielding predictions with high accordance across all different exam types of CT (47), surpassing the need for a specific calcium scoring exam.

The advances in dual-energy and photon-counting detector CT scanners furthermore suggest the use of virtual non-contrast-enhanced images from a conventional CCTA for the detection of calcification to obviate the need for additional radiation dose (48). In a recent study, dedicated PureCalcium reconstructions from CCTA outperformed CAC scoring in VNC (49). It is desirable to establish robust models for the fully automatic CAC scoring, here.

### 3.3. Coronary plaque characterization

CCTA can not only be used for the quantification of plaque burden, but also for the determination of plaque characteristics and identification of high-risk plaques, in order to aid in further treatment. Low attenuation, positive remodeling, spotty calcifications, and Napkin-ring sign are established features for the reviewing radiologist.

In clinical routine, the objective characterization of plaques apart from the expert’s visual evaluation is still lacking. It is obvious that radiomics and DL should be feasible for this task, as has been proposed in several studies. In a comparison of visual and histogram-based plaque analysis with a proposed Radiomics-based ML approach, the authors could demonstrate a higher discriminatory power for the identification of advanced atherosclerotic lesions in CCTA (50). Furthermore, it was possible to determine radiomic features that exhibit a discriminatory potential between NRS and non-NRS plaques (51) and culprit or highest-grade non-culprit lesions from lesions in stable CAD (52, 53)*.* Similarly, several studies used radiomics to distinguish vulnerable plaques in acute coronary syndrome from visually similar plaques in a population without coronary syndrome (54, 55).

The mentioned techniques currently are far from clinical implementation. Yet, these data promise great heuristic tools to raise the importance of CCTA to a new level.

### 3.4. CT-FFR

From a clinical perspective, CT-FFR is excellent because it provides insights into hemodynamics (relevance of coronary stenoses) beyond morphology. After initial very positive publications and assessments in clinical studies, the clinical value of CT-FFR and FFR measurement as a whole has recently been questioned (56). The closer the studies come to clinical practice, the more inconsistent their conclusions, and the effect of additional hemodynamic analysis seems to be relativized.

**Figure 4:**
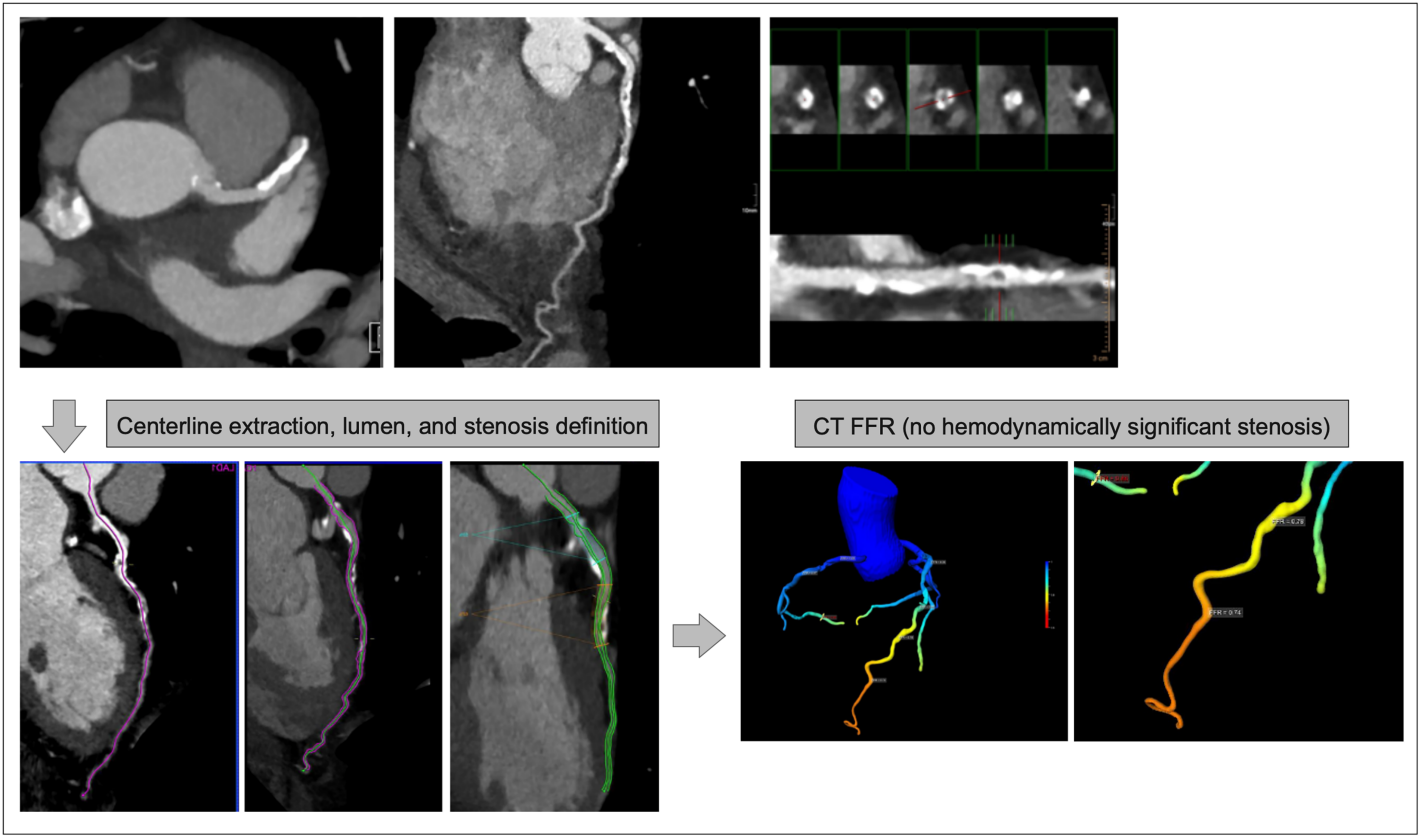
CT-FFR Case of a 78 years-old patient with significantly calcified plaques in the proximal LAD. CCTA results (upper row MIP reconstruction (left), curved (middle), and orthogonal (right) 2D-reconstructions) yielded inconclusive results concerning obstructive CAD or not; CT-FFR (lower row semiautomatic preprocessing (left), 2D/3D color-coded overview (right - LAD area magnified)) demonstrates a significant (< 0.8) reduction of hemodynamics in the LAD.

Currently, an automated determination of CT-FFR is possible by means of AI-based algorithms (57, 58), but this has so far only been certified by one provider with a time lag and considerable additional costs. For patent reasons, other ML-based algorithms offered on-site so far have only been approved for use in research settings. However, those are fast and robust and allow a rapid 3D overview of coronary hemodynamics (59). Figure 4 demonstrates the results of an ML-based CT FFR algorithm, which allows a rapid 3D overview of coronary anatomy with a color-coded annotation of hemodynmic altered coronary segments. However, these analyses are not yet integrated into the clinical workflow, and automated fused transfer to CCTA has not yet occurred.

Apart from dedicated ML and AI models trained to estimate coronary FFR, also radiomics approaches exist to predict the hemodynamic significance of coronary stenosis. For example, Wen et al. used an approach focusing on peri-coronary adipose tissue characteristics and showed, that the combination of CCTA and a decision tree radiomics model achieved significantly higher diagnostic performance (AUC: 0.812) than CCTA alone (AUC: 0.599, *p* = 0.015) for predicting the hemodynamic significance of coronary stenosis as compared to invasive FFR (60). Several studies used radiomics features to identify hemodynamically significant coronary artery stenoses, using invasive FFR as the reference standard (61, 62). Li et al. could demonstrate superiority over a conventional model trained on quantitative parameters such as plaque volume, and remodeling index (61). Denzinger et al. used plaque characterization based on DL and radiomics for predicting the revascularization decision as indicated by invasive FFR (reaching an AUC of 0.88 for a combined DL/radiomics model) (63).

### 3.5. CT perfusion

From a clinical perspective, imaging and measurement of myocardial CT perfusion are promising because they can unmask hemodynamically significant coronary stenoses that will benefit from invasive treatment. Nevertheless, the high dose requirement of current dynamic CT perfusion protocols remains problematic, thus highlighting the need for improved analysis of myocardial perfusion information from routine coronary CT angiography datasets as well as approaches that utilize AI-based algorithms to generate interpretable images from low-dose dynamic perfusion data.

Recent work by Zreik et al. (64) and van Hamersvelt et al. (65) has attempted to circumvent the need for dynamic imaging by applying DL to learn myocardial enhancement patterns associated with the presence of hemodynamically significant stenosis in epicardial coronary arteries as defined by invasive FFR. The combination of measuring the degree of luminal narrowing with DL analysis of the left ventricular myocardium in intermediate-degree coronary stenosis resulted in improved diagnostic performance for the identification of patients with functionally significant coronary artery stenosis. The proposed method resulted in improved discrimination (AUC = 0.76) compared to classification based on DS only (AUC = 0.68). The application of DL to CT image reconstruction has also led to significant improvements in the quality of low-dose CT dynamic myocardial perfusion. Takafuji et al. (66) recently proposed a DL-based method that is capable of reducing image noise by approximately 20% when compared to more conventional hybrid iterative reconstruction techniques, which potentially translates into further radiation dose savings.

Another important issue in CT dynamic perfusion imaging is the occurrence of spurious CT myocardial blood flow values due to the misregistration of temporal frames. Recent work by Lara-Hernandez et al. (67) attempted to address this issue by proposing DL-based image registration. They found their proposed method to be capable of registering dynamic cardiac perfusion sequences by reducing local tissue displacements of the left ventricle without affecting image quality, in particular the absolute CT (HU) values of the entire CT sequence. Furthermore, the DL-based approach required a much shorter processing time of a few seconds compared to conventional image registration methods.

Finally, analogous to CT-FFR and coronary calcium quantification, there is still a lack of integration of CT myocardial perfusion imaging into the clinical (structured) reporting workflow, and it is expected that AI algorithms will aid in facilitating this in the near future.

### 3.6. Scar imaging

Non-invasive detection of the presence and transmurality of myocardial scar is one of the most important applications of modern cross-sectional cardiovascular imaging. Arguably, CCTA has lagged significantly behind other modalities such as nuclear imaging techniques and most notably, cardiac magnetic resonance (CMR) imaging due to the much lower contrast between scar tissue, viable and normal myocardium. However, ML offers significant opportunities to close this gap.

Singh et al. (68) described good to excellent results utilizing CNNs to detect subendocardial scarring from delayed-enhancement CCTA scans with 91% sensitivity, 88% specificity, and 89% accuracy in comparison to human expert segmentations. Their approach consisted of combining CNN-based automated segmentation of the left ventricle with topological data analysis for geometric scar feature extraction. More recently, O’Brien et al. (69) utilized a radiomics approach for a fully-automated detection of left ventricular scarring at delayed-enhancement CCTA. Of the 93 radiomics features that were calculated, approximately two-thirds were significantly associated with the presence of myocardial scar. The 100 kV images produced the best ML classifier, a support vector machine with an AUC of 0.88. The ground truth in this study consisted of CMR-based segmentations of left ventricular scar co-registered to delayed-enhancement CT to estimate scar regions. This study provides additional proof of concept that radiomics techniques have the potential to supplement image evaluation by human experts, as well as present encouraging results of the ability of ML to make image evaluation less subjective.

Although these methods need to be further developed and validated in independent cohorts, they do provide proof of concept that delayed-enhancement CT imaging is feasible for the detection of left ventricular myocardial scarring with good accuracy.

Besides, also first radiomics approaches have been used for the detection of myocardial scars in non-contrast as well as contrast-enhanced CT imaging (70–73), which need to be validated in future studies.

## 4. Prognostic information In The epicardial adipose tissue

Epicardial adipose tissue (EAT) is recognized as a key regulator of cardiovascular health and disease (74). EAT is a metabolically active depot of visceral adipose tissue (74), and it is a biomarker of visceral (metabolically unhealthy) obesity (75). Indeed, EAT volume measured using various manual tools on CT imaging, has been related to CAD, atrial fibrillation (AF) (76), and even all-cause (including non-cardiac) mortality.

**Figure 5:**
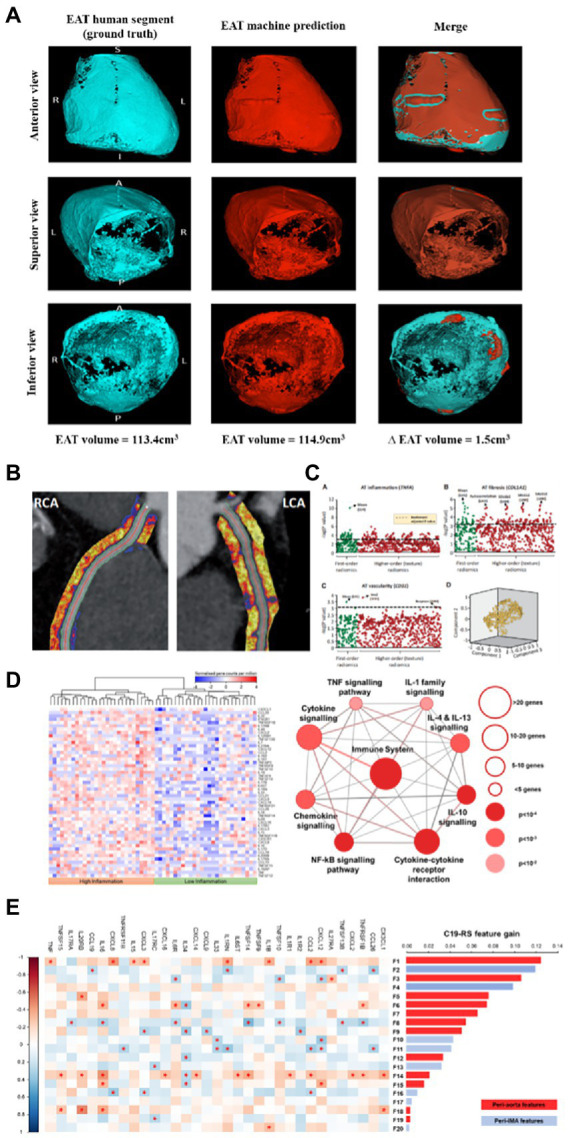
The deep learning network for automated segmentation of the EAT allows ultra-fast segmentation of the pericardium and by thresholding the adipose tissue between the pericardium and the myocardium (within the window of −30 to −190 HU) it allows accurate quantification of EAT volume **(A)**. Using a deep learning network, we can automatically segment the perivascular adipose tissue (PVAT around the coronary arteries (images from CaRi-Heart^®^ device, Caristo Diagnostics) **(B)**, from which we can extract radiomic information to best describe tissue characteristics driven by fibrosis, angiogenesis, or inflammation derived from the vascular wall **(C)**. By performing RNA sequencing on human vascular biopsies, we can generate transcriptomic signatures of different types of vascular inflammation in the vascular wall, which cause different changes in the perivascular space, depending on the degree of lipolysis, fibrosis, angiogenesis, and edema they trigger in the adjacent perivascular space **(D)**. Radiotranscriptomic phenotyping of PVAT, includes using the transcriptomic signatures established *via* RNA sequencing as the ground truth of machine learning exercises that lead to the construction of radiomic signatures to best match the transcriptomic ground truth **(E)**. Images from West et al. JACC CVI (in press) **(A)**, Oikonomou E et al. *Eur Heart J* 2019 (77) **(B,C)**, and Kotanidis C et al. *Lancet Digital Health* 2022 (78) **(D,E)**.

Although EAT volume can be estimated using various imaging modalities (ultrasound, MRI, etc.), CCTA provides the non-invasive gold standard for its quantification due to its excellent spatial resolution. Manual quantification is laborious and currently falls outside the scope of routine CCTA interpretation. In recent work, a DL approach has been applied to automate the quantification of EAT volume, with excellent precision and extremely high speed (seconds). Fully automated measurement of EAT volume incorporated into the routine interpretation of CCTA promises to significantly improve the risk stratification of patients, across several important clinical outcomes, such as predicting future atrial fibrillation (post-operative after cardiac surgery, paroxysmal or permanent in the general population), MACE and most importantly, non-cardiovascular mortality in large outcomes cohorts like SCOTHEART and ORFAN. An image with the output of such a DL network for automated measurement of EAT volume is provided in Figure 5.

Beyond EAT volume measurement, it is now known that perivascular adipose tissue (PVAT) surrounding the coronary arteries has the ability to respond to paracrine inflammatory signals from the coronary artery, activating local lipolysis and inhibiting adipogenesis in a radial way around the arterial wall (79). These 3D changes in PVAT’s composition can serve as a surrogate for vascular inflammation. A recently developed method uses automated segmentation and adipose tissue features extraction from the perivascular space to first segment PVAT around the coronary arteries, and then to quantify 3D changes in attenuation gradients corrected for several technical and local harmonization factors, allowing the calculation of the perivascular Fat Attenuation Index (FAI), a marker of coronary inflammation (79) with high prognostic value for future cardiovascular events (80, 81). This approach allows accurate prediction of cardiovascular risk in clinical practice, particularly when it is combined with information on atherosclerotic plaque burden (coronary plaque volume and composition), coronary inflammation (FAI Score for each coronary artery) and the patient’s risk factors, to calculate the patient’s absolute risk for a future cardiac event (80).

More recently, analysis of the radiomic profile of PVAT and the application of ML has led to the generation of more sophisticated biomarkers for the deep phenotyping of this adipose tissue depot (77). Different types of vascular inflammation can give different texture changes in PVAT, driven by perivascular edema, lipolysis/adipogenesis, fibrosis, and angiogenesis (74). By using tissue biopsies and RNA sequencing to generate the “ground truth” for these changes, one can train radiomic signatures of PVAT to dissect the type of vascular inflammation of interest. The field of “radiotranscriptomics” has emerged to describe the process of training radiomic signatures against the transcriptomic profile of the tissue (77). Such a radiotranscriptomic signature of PVAT analyzed from routine CCTAs, the Fat Radiomic Profile (FRP), was externally tested in the CRISP-CT (81) & SCOT-HEART cohorts (82) demonstrating the very high ability to predict future MACEs beyond traditional risk factors, coronary calcium score, coronary stenosis, and HRP features on CCTA (Figure 5) (77). A similar approach was tested successfully for detecting unstable coronary plaques from CCTA (51, 52, 83). Even more recently, a radiotranscriptomic signature of acute cytokine-driven vascular inflammation quantified in the internal mammary arteries using CCTAs or non-gated CTPAs was found to be able to detect patients with vascular inflammatory involvement, who had an 8-fold increase of their risk for in-hospital mortality and activation of systemic thrombosis after COVID-19 infection (Figure 5) (78). Such ML/radiotranscriptomic approaches, that leverage the hidden information within the transcriptome of the perivascular space, are expected to change our capacity to use PVAT as a window into vascular biology and cardiovascular risk prediction in the immediate future (see Figure 5).

## 5. Economic aspects

Despite all of the promising technical developments described above, there presently is a dearth of data evaluating the economic impact of AI techniques in routine clinical care. In general, it has been shown, that radiologists are willing to invest in AI-based assistance tools (81% investment probability in a recent study (84)). Interestingly, they preferred applications immediately supporting routine tasks like detection of abnormalities or diagnostic screening over applications that are focused on process efficiency, such as, e.g., *via* reduction of scan time (84).

Although data from cardiac imaging are still lacking, studies from other fields of imaging already hint at the potential economic impact of AI on radiology. For breast cancer detection, for example, it could be shown that the radiologist’s workload might decrease as much as by half in case commercial AI-based software assistance is used (85). This then could lead to either an overall decreased workload of the imaging specialist or – much more likely – to higher study volumes processed by the individual doctor (86). Thus, clinicians’ productivity is likely to improve when AI is used. If poorly implemented though, AI may also cause clinicians’ workload even to increase (87). Proper implementation will thus be a central aspect when developing and integrating novel AI tools into the clinical workflow.

The viewpoint of this group of authors is that AI tools need to be rigorously evaluated in clinical trials using a conceptual framework similar to that used for evaluating for example new medication. It is highly unlikely that stakeholders and especially payers will provide financial reimbursement for the use of these tools if their use does not lead to better outcomes or lower costs. Although there is a tremendous array of potential opportunities to apply AI to CCTA, it is incumbent upon the radiological and cardiovascular imaging communities to design and carry out high-quality trials to demonstrate the purported benefits. Only then will AI realize its full potential.

## 6. Legal aspects

Continuous technical progress is driving the implementation of AI into radiology practice. However, the impact of AI on the specialty is hampered by several legal and ethical hurdles. Various national regulatory authorities such as the United States Food and Drug Administration (FDA) and the European Medicines Agency (EMA) as well as many others around the world are currently developing guidance governing the clinical use of medical-grade AI applications. These regulatory agencies collaborate in the International Coalition of Medicines Regulatory Authorities (ICMRA), which sets out recommendations to help regulators address the challenges that the use of AI poses for global medicines regulation. Main recommendations include the need to apply a risk-based approach to the assessment and regulation of AI; strengthened governance structures to oversee algorithms and AI deployments, closely linked to the benefit/risk of clinically used software and medicinal products; and the development of guidelines for AI development, validation and use with medicinal products in areas such as data provenance, reliability, transparency and understandability, pharmacovigilance, and real-world monitoring of device functioning (88).

A recurring question is whether and how an AI tool can be held accountable for its activities. Since software is unlikely to be liable, the ultimate legal responsibility will rest with the human user or even developer. It is important to note that a full diagnosis often cannot be conclusively provided by an AI tool. Incomplete diagnoses, false-positive as well as false-negative predictions can cause serious errors in the treatment chain; depending on the error, a misdiagnosis can lead to economic burdens, mental stress, or – in the worst-case scenario - even the death of a patient. Provided that the error was recognizable to an expert, it is obvious that the responsibility is attributed to the user, in this case, the radiologist.

Given the current limitations of AI in terms of generalizability, bias, and accuracy the FDA, Health Canada, and the United Kingdom’s Medicines and Healthcare products Regulatory Agency (MHRA) have jointly identified 10 guiding principles that can inform the development of Good Machine Learning Practice (GMLP) (89). One of the most important core tenets of their approach to approving commercial products is that the focus should be placed on the performance of the human-AI Team. In other words, these agencies require a “human in the loop” approach in which AI algorithms are designed to function in tandem with human experts who oversee the output of AI algorithms, rather than fully autonomous AI systems that currently still suffer from serious limitations with the potential for significant clinical harm.

However, in many complex AI applications and especially in radiomics, the features that influence the prediction of the AI model are hardly recognizable by the imaging specialist anymore. A misinterpretation, for example in plaque characterization can then hardly be overruled by a human expert.

As long as it is not legally clarified who is responsible for errors caused by AI applications, its role will not go beyond that of a supporting tool, which still must be supervised by clinicians. If the responsibility for predictions made by an AI ultimately even lies with the imaging specialist, predictions made by AI will continue to be included in findings only with reservations.

## 7. Limitations

Although great progress has been made in the past decade, the presently used methods for AI development still suffer from significant shortcomings such as narrow scope which results in failure to recognize outlier cases and the potential for misdiagnosis, susceptibility to adversarial attacks, and the inability of AI to incorporate intuition, cognition and abstract reasoning. An excellent discussion of these limitations can be found in the recent special report by Ng et al. (90). These limitations need to be addressed urgently in order to clinically deploy AI with confidence.

## 8. Conclusion

AI-based algorithms for CCTA are partly already an integral part of many clinical applications and involve data acquisition, post-processing, and interpretation. In an analogous way to the setting in oncologic imaging AI-applications for CCTA are still somewhat behind. Improvements for integration of new AI-based applications into the clinical workflow are mandatory (which will vice versa increase usability and decrease costs), as well as the integration of already established structured reporting templates (CAD-RADS). The main target should be an all-in-one application for CCTA beyond specialized imaging centers.

## Author contributions

BB, MG, JH, CA, TL, and MB drafted and revised the manuscript. BB and MB reviewed the manuscript. All authors contributed to the article and approved the submitted version.

## Funding

CA acknowledges support from the British Heart Foundation (CH/F/21/90009, TG/19/2/34831, and RG/F/21/110040), the Oxford BHF Centre of Research Excellence (RE/18/3/34214), the Oxford NIHR Biomedical Research Centre and Innovate UK, Innovate UK, the European Commission, and UKRI.

## Conflict of interest

CA is the inventor of patents US10,695,023B2, PCT/GB2017/053262, GB2018/1818049.7, GR20180100490, and GR20180100510, as well as the founder, shareholder, and director of Caristo Diagnostics Ltd.

The remaining authors declare that the research was conducted in the absence of any commercial or financial relationships that could be construed as a potential conflict of interest.

## Publisher’s note

All claims expressed in this article are solely those of the authors and do not necessarily represent those of their affiliated organizations, or those of the publisher, the editors and the reviewers. Any product that may be evaluated in this article, or claim that may be made by its manufacturer, is not guaranteed or endorsed by the publisher.
